# A Dual-Specificity Inhibitor Targets Polyphosphate Kinase 1 and 2 Enzymes To Attenuate Virulence of Pseudomonas aeruginosa

**DOI:** 10.1128/mBio.00592-21

**Published:** 2021-06-15

**Authors:** Nolan Neville, Nathan Roberge, Xiang Ji, Preyesh Stephen, Jiasheng Louis Lu, Zongchao Jia

**Affiliations:** a Department of Biomedical and Molecular Sciences, Queen’s University, Kingston, Ontario, Canada; b Risen (Shanghai) Pharma Tech Co., Ltd., Shanghai, China; c Department of Biochemistry, School of Life Sciences, Fudan University, Shanghai, China; d Guangdong Key Laboratory of Nanomedicine, Chinese Academy of Sciences, Shenzhen, China; Mass General Hospital

**Keywords:** *Caenorhabditis elegans*, *Pseudomonas aeruginosa*, gallein, inhibitor, kinase, polyphosphate, virulence

## Abstract

The opportunistic pathogen Pseudomonas aeruginosa is a leading cause of nosocomial infections, which are becoming increasingly difficult to treat due to antibiotic resistance. Polyphosphate (polyP) plays a key role in P. aeruginosa virulence, stress response, and antibiotic tolerance, suggesting an attractive drug target. Here, we show that the small molecule gallein disrupts polyphosphate homeostasis by inhibiting all members of both polyphosphate kinase (PPK) families (PPK1 and PPK2) encoded by P. aeruginosa, demonstrating dual-specificity PPK inhibition for the first time. Inhibitor treatment phenocopied *ppk* deletion to reduce cellular polyP accumulation and attenuate biofilm formation, motility, and pyoverdine and pyocyanin production. Most importantly, gallein attenuated P. aeruginosa virulence in a Caenorhabditis elegans infection model and synergized with antibiotics while exhibiting negligible toxicity toward the nematodes or HEK293T cells, suggesting our discovery of dual-specificity PPK inhibitors as a promising starting point for the development of new antivirulence therapeutics.

## INTRODUCTION

Inorganic polyphosphate (polyP) is a linear polymer of inorganic phosphate residues ranging up to 1,000 monomers in length. In bacteria, polyP is synthesized and consumed by polyphosphate kinase (PPK) enzymes. These enzymes are subdivided into two families: PPK1 and PPK2. PPK1 enzymes preferentially catalyze the synthesis of polyP using ATP as a phosphodonor, while PPK2 enzymes preferentially consume polyP to phosphorylate nucleotides. Nobel laureate Arthur Kornberg first established PPK1 as a critical virulence determinant in Pseudomonas aeruginosa, demonstrating that *ppk1* knockout severely compromised quorum sensing, motility, biofilm formation, and virulence in mice (1, 2). PPK1 and PPK2 enzymes have since been implicated in the virulence and persistence of several other clinically relevant pathogens, including Escherichia coli, Campylobacter jejuni, Francisella tularensis, Mycobacterium tuberculosis, and Proteus mirabilis (3–10).

Given the importance of PPK enzymes in bacterial pathogenesis and the absence of homologous enzymes in mammals, PPKs have been lauded as potential targets for antibacterial therapeutics (1, 11). Screening efforts have discovered several inhibitors of PPK1 and PPK2 enzymes (6, 8, 12–15) (reviewed in reference 16); however, compounds with strong potency (low-micromolar or nanomolar activity) against both classes of PPKs have not yet been reported.

Inhibitors targeting both PPK1 and PPK2 enzymes would provide the most attractive therapeutic option, since many priority bacterial pathogens (e.g., P. aeruginosa, Klebsiella pneumoniae, Acinetobacter baumannii, F. tularensis, C. jejuni) possess at least one of each PPK1 and PPK2. Notably, the P. aeruginosa genome encodes three PPK2 enzymes, PPK2A (PA14_01730), PPK2B (PA14_33240), and PPK2C (PA14_19410), in addition to PPK1 (PA14_69230) (Table 1). It was recently shown that PPK2A and PPK2B of P. aeruginosa could compensate for the inactivation of PPK1, generating sufficient polyP to form granules, and in the case of PPK2A facilitate near-normal cell cycle exit (17). Thus, inhibitors with activity against both PPK classes would likely be required for complete disruption of polyP homeostasis—and downstream virulence—in P. aeruginosa and other species that possess both PPK subtypes. These findings, as well as the recent declaration by the World Health Organization of P. aeruginosa as a critical priority pathogen in urgent need of new drugs (18), led us to select this organism for our initial PPK characterization and inhibition efforts.

**TABLE 1:** PPK enzymes in P. aeruginosa

Name	PA14 gene identifier	PAO1 gene identifier	Sufficient for polyP granule formation? (17)	Preferred *in vitro* reaction (reference)
PPK1	PA14_69230	PA5242	Yes	PolyP synthesis (30)
PPK2A	PA14_01730	PA0141	Yes	Nucleoside diphosphate phosphorylation (30)
PPK2B	PA14_33240	PA2428	Yes	PolyP synthesis (this study)
PPK2C	PA14_19410	PA3455	No	Nucleoside monophosphate phosphorylation (38)

P. aeruginosa is a highly adaptable pathogen that relies upon diverse virulence mechanisms to survive in the host, prime examples of which include motility, biofilm formation, and toxin secretion (19). During the initial stages of infection, motility conferred by a polar flagellum allows P. aeruginosa to swim toward a host surface where it can attach via adhesins and begin the colonization process (20). The formation of biofilms—bacterial communities embedded in an extracellular matrix—can then follow suit. Biofilms have long been implicated in chronic P. aeruginosa infections and antibiotic resistance (21). Both planktonic and biofilm-associated P. aeruginosa also secrete a plethora of virulence factors, chief among which are pyoverdine and pyocyanin. Pyoverdine is the principal siderophore of P. aeruginosa that serves to chelate and import iron into the bacterial cell. It is also responsible for the distinctive green color of P. aeruginosa cultures. Pyocyanin is a blue-colored redox-active phenazine that causes toxicity primarily via reaction with molecular oxygen, thereby generating superoxide which damages host tissues. Interestingly pyocyanin production has been shown to increase biofilm formation (22), and biofilm formation has been shown to increase pyoverdine production (23), highlighting the interconnected nature of P. aeruginosa virulence phenotypes. The importance of these phenotypes during infection has spurred the development of numerous antivirulence strategies that seek to inhibit P. aeruginosa virulence without directly killing the bacteria (24–26).

In this study, we investigated the P. aeruginosa PPK enzymes as antivirulence targets. We recombinantly expressed and purified P. aeruginosa PPK1, PPK2A, PPK2B, and PPK2C for biochemical characterization and inhibition studies. Compound screening and analogue synthesis identified a new family of polyhydroxylated small molecules that inhibited both PPK1 and PPK2 enzymes with low-micromolar affinity, demonstrating dual-specificity inhibitors for the first time. In parallel, we uncovered a hitherto unreported role of PPK2 enzymes in P. aeruginosa virulence phenotypes, including biofilm formation, pyoverdine and pyocyanin production, and flagellar-based motility. Treatment with our dual-specificity inhibitor gallein mimicked the attenuated virulence phenotypes observed in the Δ*ppk1* Δ*ppk2A* Δ*ppk2B* Δ*ppk2C* deletion strain. Finally, we show that gallein attenuated P. aeruginosa virulence in a C. elegans model of infection and improved nematode survival. Gallein also synergized with antibiotics to protect Caenorhabditis elegans, suggesting potential clinical utility as an antibiotic adjuvant to treat resistant infections. Our work highlights PPK2 enzymes, in addition to PPK1, as important targets to disrupt P. aeruginosa polyP-mediated virulence and provides a foundation for future design of dual-specificity PPK1/PPK2 inhibitors as novel antivirulence therapeutics.

## RESULTS

### Screening identifies a new family of PPK inhibitors.

**FIG 1:**
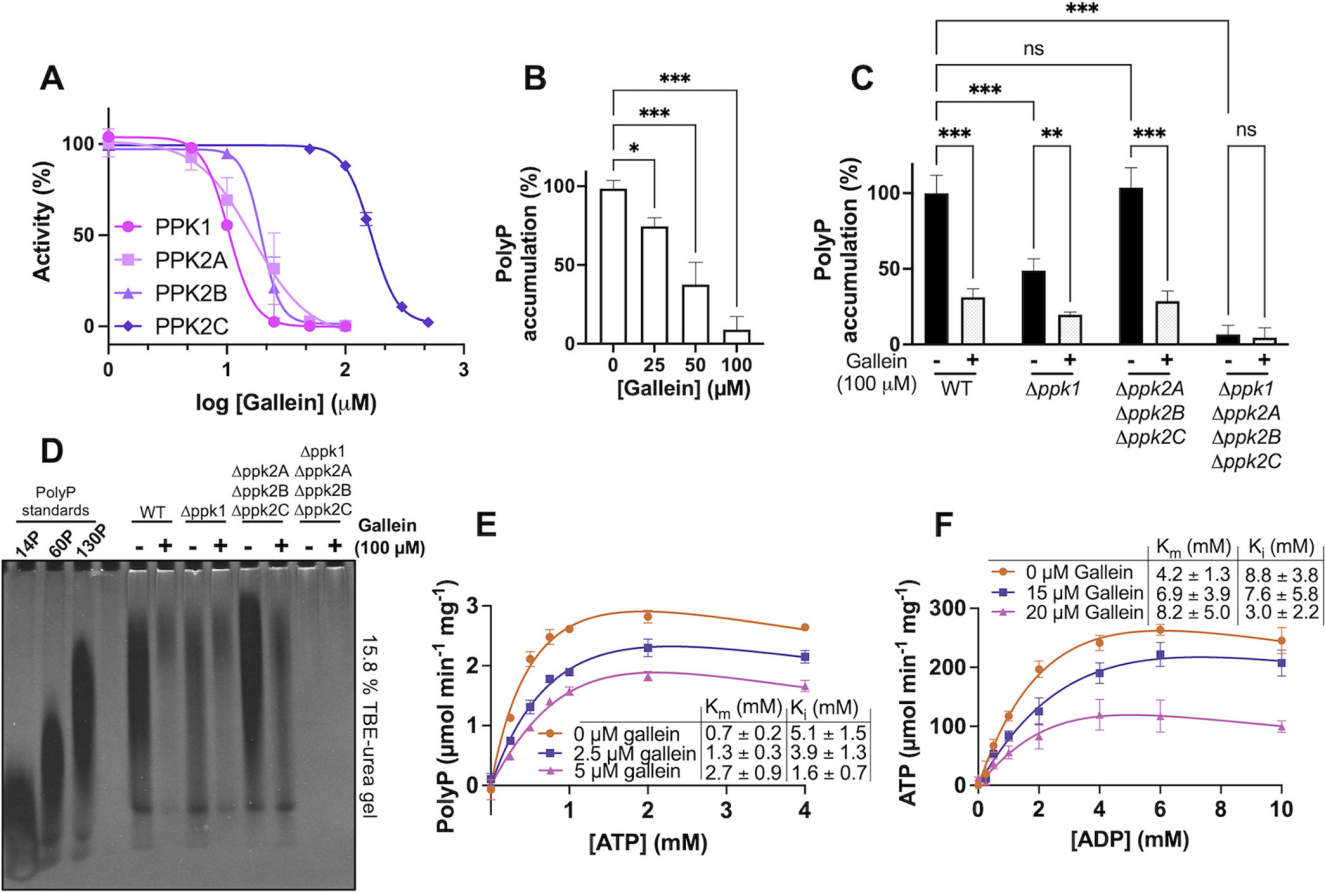
Gallein inhibits P. aeruginosa PPK1 and PPK2 enzymes *in vitro* and *in vivo*. (A) IC_50_ curves for gallein inhibition of purified PPK1 (polyP synthesis from ATP), PPK2A (ADP phosphorylation by polyP), PPK2B (polyP synthesis from ATP), and PPK2C (AMP phosphorylation by polyP) *in vitro*. (B) Dose-dependent effects of gallein on WT PA14 polyP accumulation following phosphate starvation. (C) Effect of gallein on starvation-induced polyP accumulation in PA14 strains. (D) Bacterial polyP extracts analyzed via electrophoresis on Tris-borate-EDTA (TBE)–urea gel negatively stained with DAPI. PolyP standards (14, 60, and 130 P_i_ residues) from RegenTiss are presented for chain length comparison. Image is representative of *n *= 3 gels. (E and F) Michaelis-Menten kinetics of purified PPK1 (E) and PPK2A (F) in the presence of gallein. Units represent specific activity (μmol per minute per mg PPK protein). Curves were fit with the substrate inhibition equation {*Y* = *V*_max_ × *X*/[*K_m_* + *X* × (1 + *X*/*K_i_*)]}. In panels B and C, symbols are as follows: ns, *P* > 0.05; *, *P* < 0.05; **, *P* < 0.01; ***, *P* < 0.001 (one-way analysis of variance [ANOVA] for panel B, two-way ANOVA for panel C, Tukey’s multiple-comparison test, *n *= 3). All data points are the average from triplicates; error bars are ±standard deviation.

Beginning with a small library of 116 in-house compounds from the Department of Chemistry at Queen’s University (see Data Set S1 in the supplemental material), we screened for inhibition of P. aeruginosa PPK1. Our decision to screen this library against PPK1 was motivated by the similarity of several of its compounds to ellagic acid, crude extracts of which have been previously shown to inhibit PPK1 (27). This initial screen revealed the hit compound RT1, with half-maximal inhibitory concentration (IC_50_) of 120 μM (Table 2). Based on RT1 and ellagic acid (RT3), we synthesized 45 additional analogue compounds (Data Set S1). Of these analogues, compound RT4 (commonly called gallein) yielded the greatest increase in potency with an IC_50_ of 17 μM (Table 2; Fig. 1A). Gallein was thus selected for further characterization. To assess the effects of gallein on *in vivo* PPK1 activity, we measured P. aeruginosa polyP levels following a shift to phosphate starvation medium, which is known to stimulate polyP production in bacteria (8, 28). Treatment with nonbactericidal amounts of gallein reduced wild-type (WT) polyP levels in a dose-dependent manner (Fig. 1B).

**TABLE 2:**
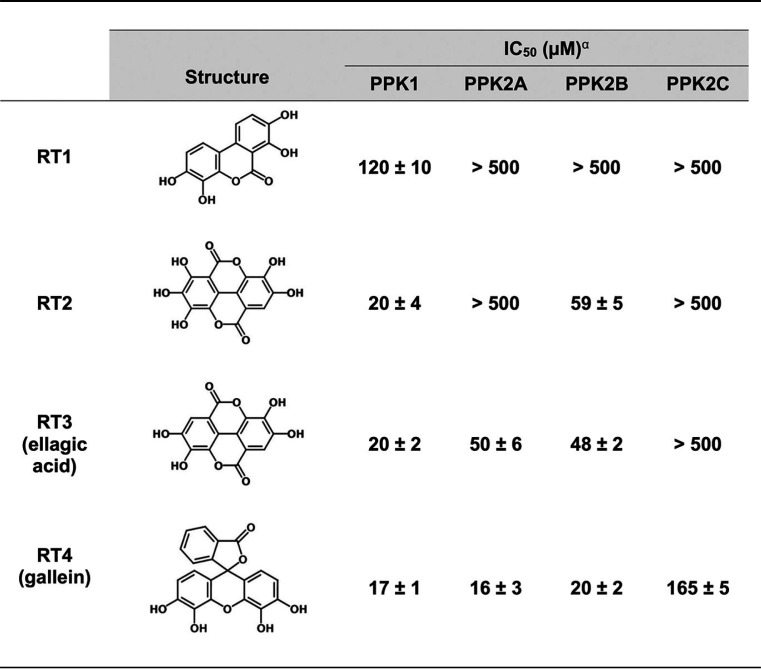
PPK inhibitor chemical structures and IC_50_ values

*^a^*IC_50_ values determined using the following reactions: PPK1, polyP synthesis from ATP; PPK2A, ATP synthesis from ADP and polyP; PPK2B, polyP synthesis from ATP; PPK2C, ADP synthesis from AMP and polyP.

10.1128/mBio.00592-21.7 DATA SET S1 Structures of chemical compounds screened. Queen’s compounds are on sheet 1, and Risen-synthesized compounds are on sheet 2. Download Data Set S1, XLSX file, 1 MB. Copyright © 2021 Neville et al. 2021 Neville et al. https://creativecommons.org/licenses/by/4.0/ This content is distributed under the terms of the Creative Commons Attribution 4.0 International license.

### Dual-specificity inhibition of PPK1 and PPK2 *in vitro* and *in vivo*.

Surprisingly, gallein treatment also significantly reduced polyP levels in the isogenic Δ*ppk1* deletion strain (Fig. 1C and D), suggesting additional targets exist for this compound in the cell. In contrast, gallein treatment had no effect on the *Δppk1 Δppk2A Δppk2B Δppk2C* quadruple knockout strain (hereafter referred to as *ΔpolyP*), the polyP levels of which remained undetectable with or without inhibitor (Fig. 1C and D). The polyP levels in the *Δppk2A Δppk2B Δppk2C* strain were comparable to those in the WT strain, and gallein treatment reduced polyP in this strain to a similar extent as that in the WT. A detailed list of comparative statistics for Fig. 1C and all subsequent figures that compare data between bacterial strains and compound treatment is available in Data Set S2. Gel electrophoresis of the polyP purified from bacterial cells qualitatively corroborated these trends, showing reduced polyP staining of samples from gallein-treated WT, *Δppk1*, and *Δppk2A Δppk2B Δppk2C* cells but not *ΔpolyP* cells (Fig. 1D). Despite accumulating less polyP overall, gallein-treated WT and *Δppk1* samples appear to contain slightly higher proportions of long-chain polyP relative to the untreated controls, while the average length of gallein-treated *Δppk2A Δppk2B Δppk2C* polyP appears to match more closely its corresponding dimethyl sulfoxide (DMSO) control. The polyP chain lengths produced by the *Δppk1* strain and the *Δppk2A Δppk2B Δppk2C* strain were similar to those produced by WT bacteria, which agrees with previous *in vitro* characterization of P. aeruginosa PPK1 and PPK2A that demonstrated these enzymes to have similar processivities (500 to 800 residues and 200 to 800 residues for PPK1 and PPK2A, respectively) (29).

**FIG 2:**
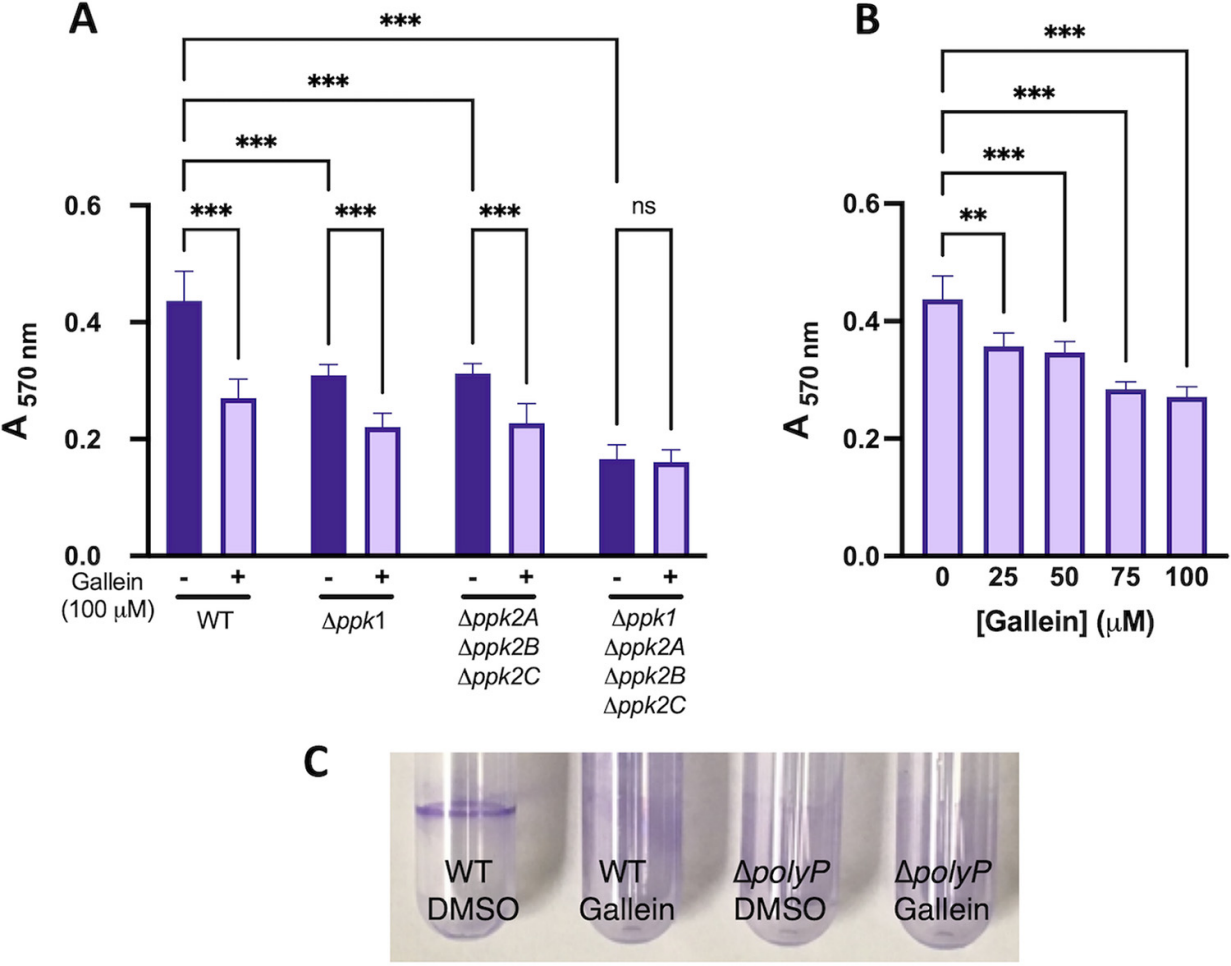
Gallein phenocopies *ppk* deletion to attenuate P. aeruginosa biofilm formation. (A) Effects of gallein treatment and *ppk* gene deletion on biofilm formation quantified via crystal violet staining. (B) Dose-dependent effects of gallein on WT PA14 biofilm formation quantified via crystal violet staining. (C) Representative image of biofilm rings. ns, *P* > 0.05; *, *P* < 0.05; **, *P* < 0.01; ***, *P* < 0.001 (two-way ANOVA for panel A, one-way ANOVA for panel B, Tukey’s multiple-comparison test, *n *= 3). All data points are the average from triplicates; error bars are ±standard deviation.

**FIG 3:**
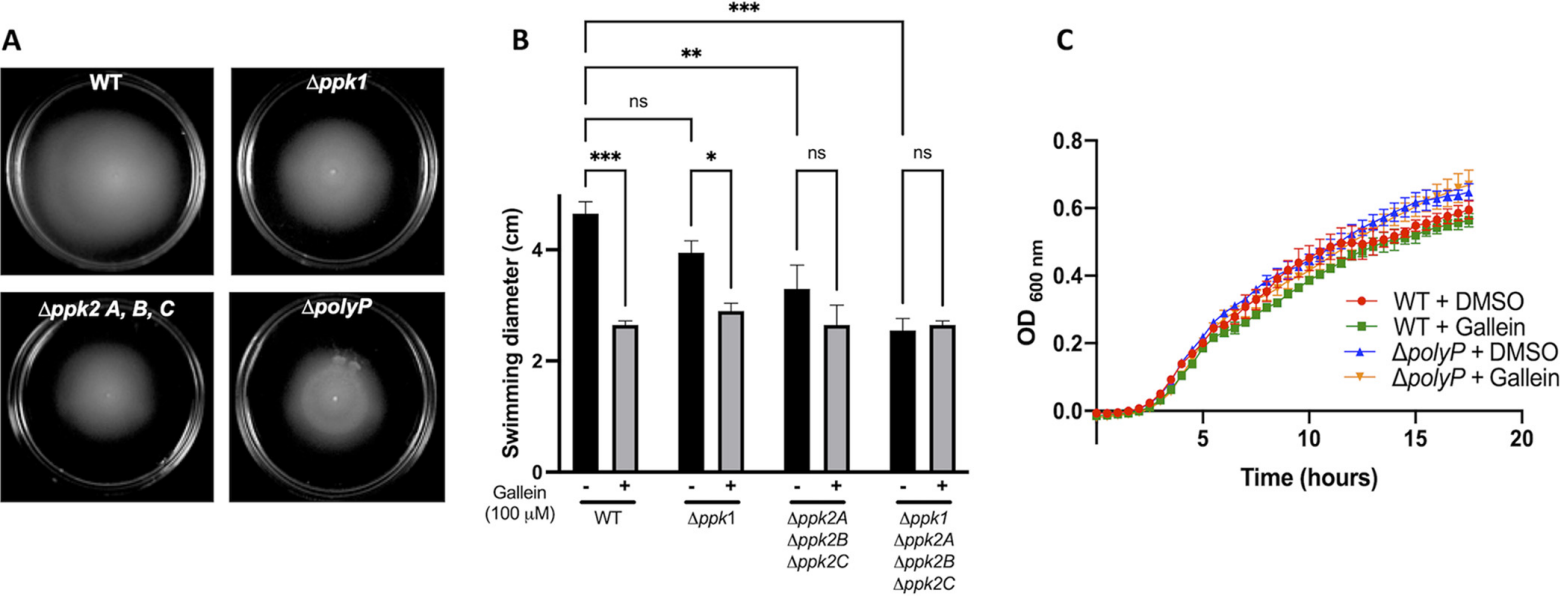
Gallein phenocopies *ppk* deletion to attenuate P. aeruginosa swimming motility. (A) Representative image of swimming motility. (B) Quantification of the effects of gallein treatment and *ppk* gene deletion on swimming motility. (C) PA14 growth kinetics in the presence and absence of gallein in lysogeny broth (equivalent in composition to swim plates, minus the agar). ns, *P* > 0.05; *, *P* < 0.05; **, *P* < 0.01; ***, *P* < 0.001 (two-way ANOVA, Tukey’s multiple-comparison test, *n *= 3). All data points are the average from triplicates; error bars are ±standard deviation.

**FIG 4:**
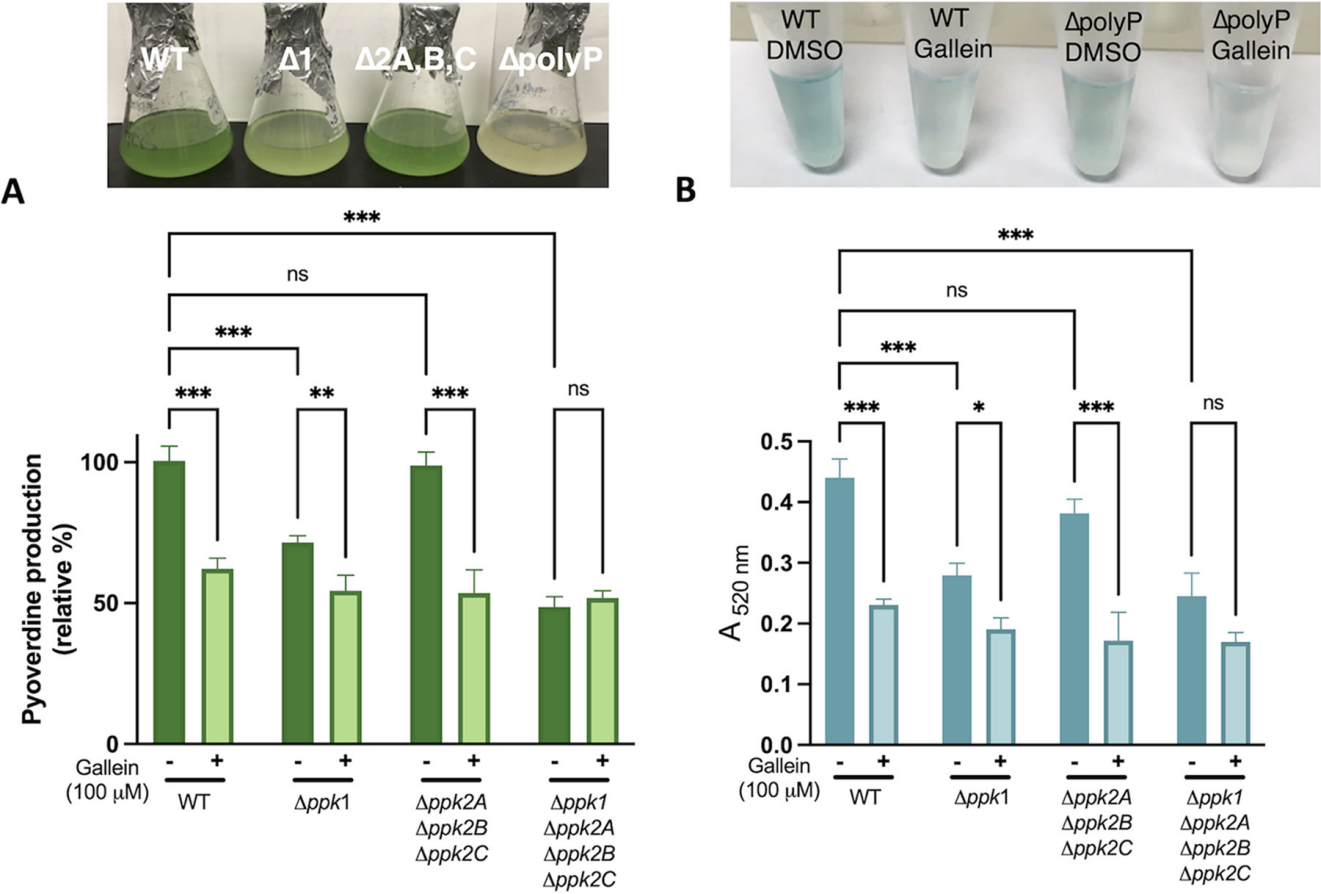
Gallein phenocopies *ppk* deletion to reduce production of toxic P. aeruginosa pigment molecules. (A) Effects of gallein treatment and *ppk* gene deletion on pyoverdine production. The top panel shows a photograph of representative cultures, and the bottom panel shows pyoverdine quantification via absorbance at 403 nm. (B) Effects of gallein treatment and *ppk* gene deletion on pyocyanin production. The top panel shows a photograph of representative pyocyanin extracts, and the bottom panel shows pyocyanin quantification via absorbance at 520 nm following chloroform extraction and HCl treatment. ns, *P* > 0.05; *, *P* < 0.05; **, *P* < 0.01; ***, *P* < 0.001 (two-way ANOVA, Tukey’s multiple-comparison test, *n *= 3). All data points are the average from triplicates; error bars are ±standard deviation.

10.1128/mBio.00592-21.8 DATA SET S2 Comprehensive tables of comparative statistics for Fig. 1C, 2A, 3B, 4A and B, and 5D and E. Download Data Set S2, XLSX file, 0.02 MB. Copyright © 2021 Neville et al. 2021 Neville et al. https://creativecommons.org/licenses/by/4.0/ This content is distributed under the terms of the Creative Commons Attribution 4.0 International license.

In P. aeruginosa, PPK2 enzymes can compensate for the loss of PPK1, allowing Δ*ppk1* bacteria to accumulate reduced, but detectable, levels of polyP (17, 30). The additive effects of gallein on the Δ*ppk1* strain, coupled with the absence of activity on the *ΔpolyP* strain, led us to hypothesize that gallein was also inhibiting PPK2 enzymes. To test this, we expressed and purified recombinant PPK2A, PPK2B, and PPK2C and assayed the effects of our inhibitors. Interestingly, recombinant PPK2B showed a strong preference for polyP synthesis *in vitro* in the presence of an ATP regeneration system (see Text S1), in contrast to PPK2A and PPK2C (Table 1; Fig. S1B). Gallein inhibited purified recombinant PPK2A and PPK2B at low-micromolar concentrations (Fig. 1A; Table 2). Inhibition of PPK2C was less dramatic (IC_50_ = 165 μM) and likely contributes minimally to polyP levels *in vivo* given that PPK2C alone is insufficient for polyP granule biogenesis (17). To rule out the possibility that gallein was aggregating as a pan-assay interference compound (PAIN), we repeated *in vitro* enzyme inhibition assays of all four PPK enzymes in the presence of 0.01% (vol/vol) Triton X-100 (31). Negligible change in IC_50_ values indicated that gallein was not an aggregation PAIN (Fig. S2).

10.1128/mBio.00592-21.1 TEXT S1 Supplementary notes and discussion. Download Text S1, DOCX file, 0.1 MB. Copyright © 2021 Neville et al. 2021 Neville et al. https://creativecommons.org/licenses/by/4.0/ This content is distributed under the terms of the Creative Commons Attribution 4.0 International license.

10.1128/mBio.00592-21.2 FIG S1 *In vitro* polyphosphate synthesis activity and inhibition by gallein. (A) Toluidine blue standard curve. PolyP concentrations are in terms of P_i_ monomers. (B) PolyP synthesis rates of recombinant P. aeruginosa PPK enzymes. A 275 nM concentration of each enzyme was added to 100-μl reaction mixtures that contained creatine kinase and creatine phosphate for ATP regeneration. Reactions were stopped after a 10-minute incubation at 37°C. (C) PPK1 polyP synthesis activity rate in the absence of ATP regeneration (no creatine kinase or creatine phosphate). Reaction mixtures were incubated at 37°C for 1 h. (D) PPK1 polyP synthesis reaction mixtures without ATP regeneration (no creatine kinase or creatine phosphate) were incubated at 37°C overnight to reach equilibrium. In panels C and D, reaction mixtures (100 μl) contained 275 nM PPK1, 50 mM HEPES-KOH (pH 7.5), 40 mM (NH_4_)_2_SO_4_, 4 mM MgSO_4_, and 5 mM ATP. For each panel, *n *= 3; values are means ± standard deviation. Download FIG S1, TIF file, 2.7 MB. Copyright © 2021 Neville et al. 2021 Neville et al. https://creativecommons.org/licenses/by/4.0/ This content is distributed under the terms of the Creative Commons Attribution 4.0 International license.

10.1128/mBio.00592-21.3 FIG S2 Counterscreen for aggregation PAINs. PPK1 reaction mixture was treated with 50 μM (each) inhibitor, with (+) or without (−) the addition of Triton X-100 to a 0.01% final concentration. PPK1 inhibition was recorded for each sample, and the inhibition of the Triton-free sample was set to 100%. Error bars ± standard deviation. Download FIG S2, TIF file, 2.4 MB. Copyright © 2021 Neville et al. 2021 Neville et al. https://creativecommons.org/licenses/by/4.0/ This content is distributed under the terms of the Creative Commons Attribution 4.0 International license.

### Gallein treatment phenocopies *ppk1* and *ppk2* deletion.

Given the well-documented dependence of biofilm formation on PPK1 (1, 8), we tested the effects of gallein on this phenotype in P. aeruginosa. As expected, gallein treatment reduced biofilm formation (Fig. 2A and C), and this reduction was dose dependent (Fig. 2B). Treatment of the Δ*ppk1* strain further decreased biofilm formation, as did treatment of the Δ*ppk2* strain. The Δ*polyP* strain formed the least biofilm, and gallein treatment had no significant effect on this strain. Gallein did not affect the planktonic growth rate of P. aeruginosa under the biofilm assay conditions (Fig. S4A). Gallein treatment also faithfully reproduced in WT P. aeruginosa the defect in flagellum-based swimming motility observed in the Δ*polyP* strain (Fig. 3A and B). The Δ*ppk2* strain also exhibited significant swimming defects, indicating for the first time the involvement of PPK2 enzymes in this virulence phenotype. Gallein treatment did not affect bacterial growth kinetics (Fig. 3C), ruling out the possibility of slowed growth as a variable in the swimming phenotype. Finally, we demonstrated the involvement of PPK1 and PPK2 enzymes in the production of pyoverdine and pyocyanin. The green-colored siderophore pyoverdine is secreted by P. aeruginosa during stationary phase to chelate and subsequently import iron—a key nutrient that is often growth limiting during infection (33). Pyocyanin is a blue-colored phenazine molecule that is primarily associated with oxidative damage of host cells. The Δ*ppk1* strain showed a 40 to 50% reduction in pyoverdine and pyocyanin production, while the Δ*ppk2* strain showed only slight defects (Fig. 4A and B). However, levels of pyoverdine produced by the Δ*polyP* strain were further reduced by approximately 20% relative to the Δ*ppk1* strain. Thus, the role of PPK2 enzymes in pyoverdine production may become fully apparent only in cells lacking PPK1 activity. Treatment of the WT, Δ*ppk1*, and Δ*ppk2* strains with gallein reproduced the marked pyoverdine and pyocyanin defects observed in the Δ*polyP* strain, while treatment of the Δ*polyP* strain yielded no significant decrease of either toxin (Fig. 4A and B).

10.1128/mBio.00592-21.5 FIG S4 Growth curves of P. aeruginosa WT and Δ*polyP* in M63 biofilm medium (A) and liquid killing C. elegans assay medium (B). Gallein, or an equivalent volume of DMSO, was added to a 100 μM final concentration. OD_600_ monitored in triplicate cultures grown in 96-well plates at 37°C following colony inoculation. Error bars ± standard deviation. Download FIG S4, TIF file, 2.5 MB. Copyright © 2021 Neville et al. 2021 Neville et al. https://creativecommons.org/licenses/by/4.0/ This content is distributed under the terms of the Creative Commons Attribution 4.0 International license.

### Validation of gallein antivirulence activity in a C. elegans infection model.

**FIG 5:**
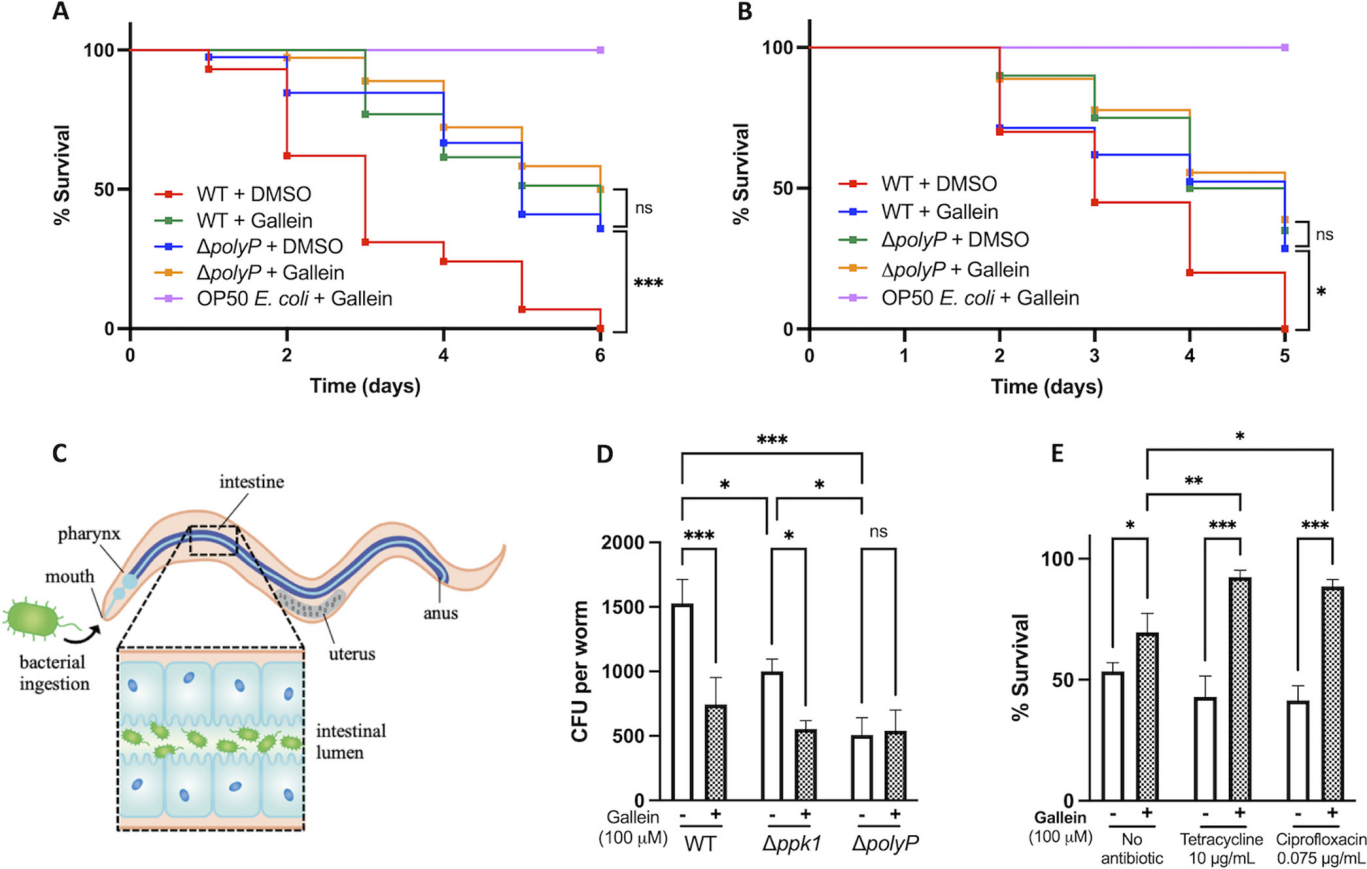
Gallein attenuates P. aeruginosa virulence in C. elegans infection models. (A and B) Kaplan-Meier plot of liquid killing (A) and solid killing (B) assays. Where indicated, gallein was added to 100 μM final concentration. (C) Schematic of bacterial accumulation in the nematode gut. (D) Enumeration of P. aeruginosa CFU to quantify bacterial accumulation in the C. elegans gut. (E) Gallein synergizes with antibiotics to potentiate their activity at sub-MIC doses. (A and B) *, *P* < 0.05; ***, *P* < 0.001 (Mantel-Cox log rank test, *n *= 30 worms per condition). (D and E) ns, *P* > 0.05; *, *P* < 0.05; **, *P* < 0.01; ***, *P* < 0.001 (two-way ANOVA, Tukey’s multiple-comparison test, *n *= 30 worms per condition).

Having demonstrated the ability of gallein to reduce virulence phenotypes in cultured P. aeruginosa cells, we tested whether this inhibitor could achieve a similar attenuation of virulence in C. elegans. These nematodes are widely used as models for P. aeruginosa pathogenesis, and PPK1 activity has been shown to be important for C. elegans colonization by P. aeruginosa (8). We tested both liquid killing (LK) and solid-phase slow killing (SK) models of infection, as these two assays are reliant upon distinct mechanisms of P. aeruginosa pathogenesis (34, 35). In both LK and SK, gallein treatment significantly improved survival rates, mirroring the survival benefit observed in nematodes infected with the Δ*polyP* strain (Fig. 5A and B). Gallein-treated and Δ*polyP* bacteria exhibited near-WT growth kinetics under LK conditions (Fig. S4B), indicating that the effects on C. elegans infection were not a result of attenuated bacterial growth. Notably, no gallein toxicity toward C. elegans was observed at the 100 μM concentration used in these assays (OP50 + gallein curves). Gallein treatment also phenocopied the Δ*polyP* strain and reduced bacterial accumulation in the nematode gut (Fig. 5C and D). Treatment of the *Δppk1* strain further reduced bacterial accumulation, while treatment of the Δ*polyP* strain yielded no additional decrease in CFU. Gallein also synergized with antibiotics in the LK assay, whereby gallein in combination with either tetracycline or ciprofloxacin achieved greater protection of C. elegans than did gallein or antibiotics alone (Fig. 5E). Gallein (100 μM) did not alter the MIC of tetracycline (16 μg/ml) or ciprofloxacin (0.125 μg/ml) in LK medium, suggesting that the mechanism of synergy is distinct from typical antibiotic killing.

### Gallein avoids mammalian kinase toxicity.

**FIG 6:**
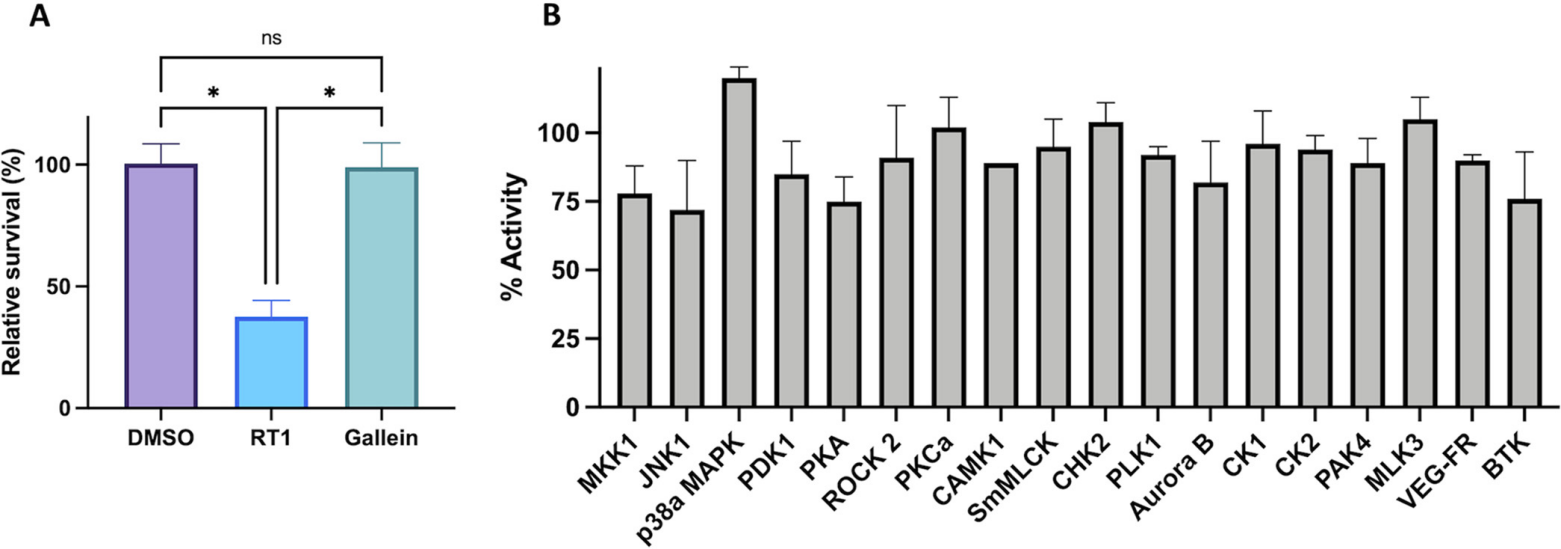
Gallein avoids off-target toxicity. (A) HEK cell cytotoxicity assay. Viability determined via trypan blue staining following 48 h of incubation with compounds. (B) Counterscreen for inhibition of human protein kinases by 10 μM gallein via radioactive ([^33^P]ATP) filter-binding assay performed by the University of Dundee MRC PPU (26). (A) ns, *P* > 0.05; *, *P* < 0.05 (one-way ANOVA, Tukey’s multiple-comparison test, *n *= 3).

Toxicity is always a major concern with kinase inhibitors, even though the absence of PPK homologues in mammals makes these enzymes particularly attractive therapeutic targets (1, 11). Cytotoxicity of the inhibitors was therefore assessed in cultured human embryonic kidney (HEK) 239T cells via standard trypan blue viability assays (36). While the initial hit RT1 reduced cell viability by ∼60%, gallein was noncytotoxic at concentrations up to 100 μM (Fig. 6A). Gallein was also counterscreened against an array of divergent human kinases available at the International Centre for Kinase Profiling (University of Dundee), as described previously (37). Off-target inhibition of these kinases was negligible, with no greater than 25% loss of activity in the presence of gallein (Fig. 6B).

## DISCUSSION

Twenty years ago, pioneering work by the late Nobel laureate Arthur Kornberg first established the importance of polyP for P. aeruginosa virulence. A P. aeruginosa knockout mutant in the *ppk1* gene was aberrant in quorum sensing-mediated toxin secretion, biofilm formation, and motility (1, 2). Most remarkably, the *ppk1* mutant also displayed attenuated virulence in a burned-mouse model of infection (1). Noting that their P. aeruginosa *ppk1* mutant still synthesized up to 20% of WT polyP levels (2, 30), the authors’ further investigation identified the enzyme that was later called PPK2A as the source of this polyP—thereby establishing PPK2s as a novel enzyme class present in P. aeruginosa and other bacteria (29). While PPK2A (30), and later PPK2C (38), has been biochemically characterized, the collective involvement of PPK2 enzymes in P. aeruginosa physiology and virulence had never been investigated. Spurred by the recent findings that PPK2A and PPK2B can compensate for the loss of PPK1 to form polyP granules (17), we sought to investigate the roles of PPK2 enzymes in P. aeruginosa virulence and explore their potential in conjunction with PPK1 as antivirulence drug targets.

**FIG 7:**
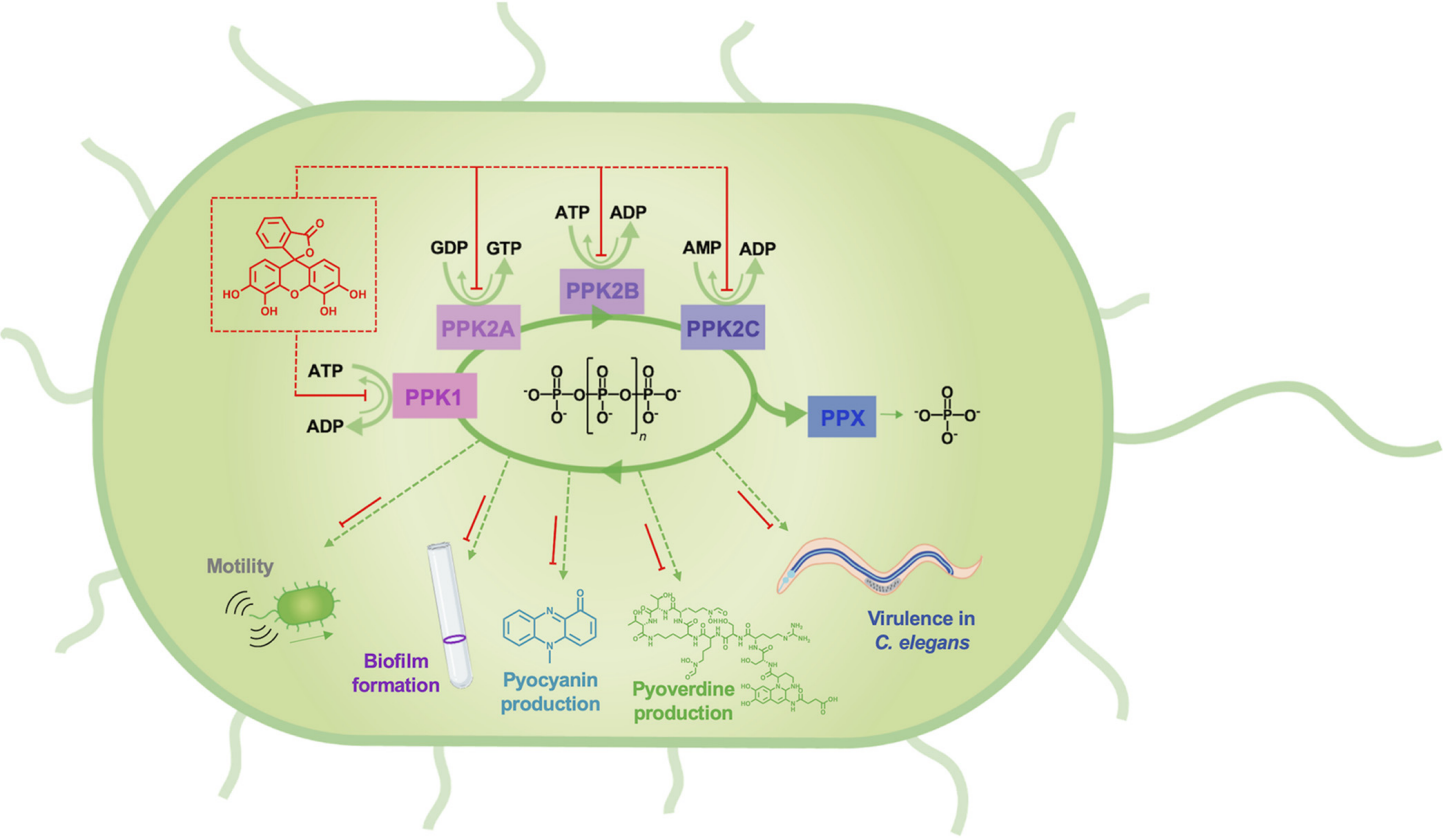
Schematic summary of P. aeruginosa PPK enzyme inhibition by gallein. Gallein inhibits PPK1, PPK2A, PPK2B, and PPK2C to disrupt polyP homeostasis, which results in downstream attenuation of motility, biofilm formation, pyoverdine and pyocyanin production, and virulence toward C. elegans.

Previous studies have documented several PPK1 (8, 12, 13) and PPK2 (6, 14, 15) inhibitors, but lacking has been a molecule capable of potently inhibiting both PPK1s and PPK2s. Thus, development of a dual-specificity inhibitor that acts on both classes of enzymes would be an attractive approach. Using ellagic acid and RT1 as a starting point (27), our synthesis of related compounds yielded the hit compound gallein that inhibited PPK1, PPK2A, and PPK2B at low-micromolar concentrations (Fig. 7). While PPK1 and PPK2 enzymes are capable of catalyzing the same chemical reactions, they share negligible sequence similarity or structural homology (29). PPK1 enzymes are large (∼75 kDa), share structural similarity with the phospholipase D family of proteins, and catalyze reactions via a phosphohistidine intermediate (39). In contrast, PPK2 enzymes are smaller (∼35 kDa) and share structural and mechanistic similarity with thymidylate kinases, whereby Walker A and B motifs position the nucleotide substrate for Mg^2+^-catalyzed nucleophilic attack on the terminal phosphate of polyP (38, 40). Despite these differences, our kinetic and docking data suggest that gallein acts as a nucleotide mimetic to bind in the active sites of both enzyme classes. The weaker inhibition by gallein of PPK2C may be explained by the fact that this enzyme is a class II PPK2—catalyzing the conversion of nucleoside monophosphates into nucleoside diphosphates—while PPK2A and -B are class I enzymes that preferentially phosphorylate nucleoside diphosphates (40). Indeed, structural comparison of F. tularensis PPK2 (class I) and Meiothermus ruber PPK2 (class III; can phosphorylate either nucleoside mono- or diphosphates) revealed distinct nucleotide binding orientations, with the adenosine moiety being shifted ∼4.5 Å deeper into the *M. ruber* protein (40). Alternatively, the extra noncatalytic domain of PPK2C (38) may have an impact on its active site such that gallein binding is less favorable. We note the structural similarity between gallein and NSC 9037, a previously described inhibitor of M. tuberculosis PPK2 (6), which may suggest that gallein-based inhibitors could be broadly applicable to combat other *ppk2*-carrying bacterial pathogens.

The dual specificity of gallein proved effective at attenuating P. aeruginosa virulence phenotypes. The Kornberg group had hypothesized that PPK2A was important for P. aeruginosa biofilm formation due to its ability to synthesize GTP, which is required for downstream alginate production—a key component of pseudomonad biofilms (30). Indeed, we provide the first cell-based evidence that *ppk2* deletion, or PPK2 inhibition by gallein, reduces biofilm formation. Biofilms are intimately linked to chronic P. aeruginosa infection and antibiotic resistance (21), highlighting the utility of disrupting this phenotype. Similarly, the siderophore pyoverdine and the redox-active phenazine pyocyanin underpin P. aeruginosa virulence and clinical outcomes (41, 42). Gallein reduced pyoverdine and pyocyanin to nearly *ΔpolyP* levels. This marks the first direct evidence of PPK involvements in pyoverdine and pyocyanin production, although exopolyphosphatase PPX was previously implicated in the production of both toxins (43). Given the role of pyoverdine as an iron-importing siderophore, it is intriguing to note the recent discovery that polyP serves as an iron chelator and Fenton reaction inhibitor in E. coli (44). It is thus tempting to speculate that the reduced pyoverdine production observed in Δ*polyP* and gallein-treated bacteria may serve as a mechanism to avoid accumulating toxic levels of intracellular iron in the absence of polyP-mediated iron storage capacity. Future studies should investigate potential mechanistic links between iron levels, polyP levels, and siderophores in P. aeruginosa and other bacterial species.

Flagellar motility, which is key for establishing acute infections (20), was also compromised by gallein treatment in a *ppk*-dependent fashion, corroborating previous reports linking polyP to this phenotype in P. aeruginosa (2, 43). Once again, our results show that deletion of all four *ppk* genes, or treatment with gallein, attenuates motility more than deletion of *ppk1* alone. Importantly, no significant difference in growth kinetics was observed between WT, *ΔpolyP*, or gallein-treated cells under any of the medium conditions used for these assays (Fig. 3C; see also Fig. S4 in the supplemental material). This rules out potential differences in cell density, or general bactericidal effects of the inhibitors, as confounding variables in our phenotypic analyses. The absence of additive effects of gallein treatment on *ΔpolyP* cells during any of the described virulence assays strongly suggests that PPK enzymes are indeed the targets of this compound in the cell.

The value of inhibiting both PPK1 and PPK2 enzymes was further supported in C. elegans models of infection. In both LK and SK assays, gallein treatment of WT P. aeruginosa phenocopied the attenuated virulence of the *ΔpolyP* strain. In LK, P. aeruginosa pathogenesis is primarily mediated by pyoverdine (35). Thus, attenuated LK virulence corroborates the reduced pyoverdine levels observed in gallein-treated and *ΔpolyP* cells (Fig. 4A). In contrast, SK involves bacterial accumulation inside the nematodes and requires intact quorum sensing pathways for full lethality (34). Indeed, we also observed reduced accumulation of polyP-deficient bacteria in the C. elegans gut, as seen in previous reports (8). Given the disparate mechanisms involved in LK and SK, the attenuated virulence of gallein-treated and *ΔpolyP* bacteria in both assays underlines the pleiotropic role of PPK enzymes in P. aeruginosa virulence. Combination of gallein with the antibiotic tetracycline or ciprofloxacin resulted in synergetic protection of C. elegans in LK. Notably, this synergy occurred at sub-MIC quantities of the antibiotics, suggesting that PPK inhibitors such as gallein could be used to potentiate the activity of antibiotics which would otherwise be considered unusable in the clinic.

It is worth noting that gallein has been previously shown to inhibit mammalian G protein-coupled receptors (GPCRs) (45) and that C. elegans encodes a GPCR called OCTR-1 which is known to play a role in innate immunity (46) and could ostensibly confound our assays if it too was a target of gallein. However, the absence of any additive survival benefit upon gallein treatment of C. elegans infected with the Δ*polyP* P. aeruginosa strain—in contrast to the WT strain—strongly argues against this possibility since off-target OCTR-1 inhibition would be expected to further extend the C. elegans life span in these samples if it were conferring a generalized immune advantage. Importantly, gallein showed no detectable toxicity toward either C. elegans or cultured mammalian cells. This corroborates previous studies in which gallein was administered at 5 to 10 mg/kg of body weight daily to mice and rats for up to 8 weeks with no adverse effects (47–49). Our work therefore provides a molecule with a favorable toxicity profile as a starting point for the development of dual-specificity PPK1 and PPK2 inhibitors.

While this work focused solely on P. aeruginosa strain PA14 for preliminary testing, it is important to note that the genes encoding PPK1, PPK2A, PPK2B, and PPK2C are present in all P. aeruginosa genomes, including sequenced clinical isolates. More broadly, PPK1 and PPK2 orthologues are also present in numerous other human pathogens such as A. baumannii, K. pneumoniae, and M. tuberculosis (29). Given the highly conserved nature of PPK1 and PPK2 enzymes, it is reasonable to assume gallein may also inhibit these enzymes in other bacteria. With a dwindling arsenal of antibiotics available to treat bacterial infections (50), our dual-specificity PPK1 and PPK2 inhibitor may thus have the potential to become a useful antivirulence therapeutic against P. aeruginosa and other pathogens. By providing direct evidence of PPK2 enzyme involvement in P. aeruginosa virulence phenotypes, this work also establishes PPK2s, in addition to PPK1, as valuable targets for future drug design efforts and studies of P. aeruginosa polyP physiology.

## MATERIALS AND METHODS

### Strains and growth conditions.

All strains and plasmids used in this study are listed in Table S1 in the supplemental material. P. aeruginosa UCBPP-PA14 and its various *ppk* deletion derivatives were provided by L. Racki (Scripps Research) (17). Unless otherwise specified, P. aeruginosa was cultured aerobically at 37°C in lysogeny broth (LB). C. elegans worms were maintained at 25°C (N2 WT) or 16°C (*glp-4* mutants) on nematode growth medium agar plates seeded with E. coli OP50.

10.1128/mBio.00592-21.6 TABLE S1 Strains, plasmids, and oligonucleotides used in this study. Download Table S1, DOCX file, 0.02 MB. Copyright © 2021 Neville et al. 2021 Neville et al. https://creativecommons.org/licenses/by/4.0/ This content is distributed under the terms of the Creative Commons Attribution 4.0 International license.

### Cloning, heterologous expression, and purifications of proteins.

The codon-optimized P. aeruginosa *ppk1* gene cloned into a pET-TEV expression vector was provided by F. Chávez (Universidad de Chile) and expressed as described previously (12). PPK1 protein was membrane extracted as described previously (51) and then purified via Ni^2+^-nitrilotriacetic acid (NTA) affinity. Elution fractions were dialyzed overnight at 4°C in the presence of 0.02 mg/ml His-tagged tobacco etch virus (TEV) protease into buffer containing 20 mM Tris-HCl (pH 8.5), 200 mM NaCl, 15% glycerol, and 5 mM β-mercaptoethanol (β-ME). The mixture was then applied to a fresh Ni^2+^-NTA column to remove TEV and the cleaved His tag. Purified PPK1 was stored in liquid nitrogen. Gene sequences corresponding to PPK2A (stable truncation; residues P9 to P289), PPK2B, and PPK2C were amplified from P. aeruginosa PA14 genomic DNA via PCR using the primers in Table S1. The fragments were then restriction digested (BamHI and XhoI for PPK2A and PPK2B; BamHI and EcoRI for PPK2C) and subsequently ligated into a modified pET16b vector (HT29) to yield an N-terminal maltose binding protein (MBP) and His tag fusion separated from the protein of interest by a TEV cleavage site. Recombinant plasmids (Table S1) were introduced into BL21(DE3) competent cells for protein expression with isopropyl-β-d-thiogalactopyranoside (IPTG). Harvested cells were resuspended in lysis buffer (50 mM HEPES-Na, pH 7.5, 300 mM NaCl, 2 mM β-ME, and 5% glycerol) and sonicated for lysis. Lysate was clarified via centrifugation at 18,000 rpm for 30 min and then applied to Ni^2+^-NTA resin. Proteins were eluted with a 0 to 300 mM gradient of imidazole. Eluted MBP-tagged proteins were subjected to overnight TEV protease (0.02 mg/ml) digestion at 4°C while dialyzing against lysis buffer. Digested samples were loaded onto amylose resin to remove the MBP tag, and the flowthrough that contained PPK was concentrated and loaded onto a Superdex 200 column (GE Healthcare) for size exclusion chromatography using lysis buffer. The pooled fractions were flash frozen in liquid nitrogen and stored at −80°C.

### Compound synthesis and screening.

Compounds were synthesized as outlined in Text S1. Compounds were dissolved in DMSO to make 10 mM stocks and added to a 200 μM final concentration for initial screening. The screen for PPK1 inhibitors was conducted by measuring polyP synthesis activity as described previously (12), with the exception of the polyP detection method (see Text S1). The reaction mix contained 50 mM HEPES-KOH, pH 7.5, 40 mM (NH_4_)_2_SO_4_, 4 mM MgSO_4_, 60 mM creatine phosphate, 0.0625 mg/ml creatine kinase (Sigma; from rabbit muscle), 5 mM ATP, and 275 nM PPK1 (added last). One-hundred-microliter reaction mixtures were incubated at 37°C for 30 min, after which time 50-μl aliquots were withdrawn and added to 1,000 μl of 6-mg/liter toluidine blue dissolved in 40 mM acetic acid to quench the reaction and quantify polyP (27). The *A*_630/530 nm_ ratio was then recorded, and the values obtained were corrected by subtracting the corresponding blanks (all ingredients except enzyme). The amount of polyP synthesized was calculated using a standard curve (Fig. S1A). Any compounds showing 50% or greater reduction in activity relative to the DMSO control were subjected to further IC_50_ analysis.

### IC_50_ and kinetic assays.

All IC_50_ and kinetic assay mixtures contained equimolar amounts (275 nM) of PPK to ensure valid comparison between different enzymes. PolyP synthesis activity (for PPK1 and PPK2B) was assayed using the reaction buffer and conditions described above. ATP concentration was varied as indicated for kinetic experiments. Curves were fit using GraphPad Prism V9.

Nucleotide phosphorylation activity of PPK2 enzymes was assayed in buffer containing 50 mM Tris-HCl, pH 7.5, 10 mM (NH_4_)_2_SO_4_, 10 mM MgCl_2_, 5 mM polyP_45_ (Sigma; in terms of individual P_i_ monomers), and nucleotide (ADP or AMP) at 5 mM for IC_50_ reactions or as indicated for kinetic experiments. Enzyme was added to a final concentration of 275 nM in 100-μl reaction volumes. One-hundred-microliter reaction mixtures were incubated at 37°C for 30 min, and then 5-μl aliquots were withdrawn and added to 1,000 μl of 6-mg/liter toluidine blue dissolved in 40 mM acetic acid to quench the reaction and quantify the polyP that remained. The *A*_630/530 nm_ ratio was then recorded, and the values obtained were corrected by subtracting the corresponding blanks (all ingredients except enzyme). The amount of polyP consumed was calculated by subtracting the amount present after reaction from that present in the initial reaction mix, as calculated via a standard curve. Each monomer of P_i_ consumed from polyP equates to one nucleoside phosphorylated. Curves were fit using GraphPad Prism V9.

### Bacterial polyP accumulation assay.

P. aeruginosa polyP levels following nutrient deprivation in morpholinepropanesulfonic acid (MOPS) minimal medium were assessed as described previously (8), with slight variations. Briefly, overnight cultures were grown in 5 ml LB in the presence of inhibitor or DMSO as indicated. Cultures were centrifuged for 10 min at 3,900 rpm to pellet cells, which were then resuspended in MOPS minimal medium containing 100 μM phosphate and the indicated quantity of inhibitor or DMSO. Cultures were incubated at 37°C for 2 h to allow polyP accumulation and then subsequently pelleted, resuspended in guanidinium thiocyanate (GITC) lysis buffer, and boiled at 95°C for 5 min. PolyP was purified via silica spin column as described previously (8), with the exception of using 50 nM PPK2A instead of E. coli PPK to convert polyP to ATP. An Invitrogen ATP determination kit (ThermoFisher) was used per the manufacturer’s instructions for ATP quantification. Luminescence was recorded on a SpectraMax iD3 microplate reader (Molecular Devices).

### PolyP electrophoresis.

PolyP samples were extracted and purified via silica spin column as described above. The purified eluate was then mixed with loading dye (10 mM Tris-HCl, pH 7, 1 mM EDTA, 30% glycerol, bromophenol blue) and electrophoresed on 15.8% Tris-borate-EDTA (TBE)–urea gels as described previously (52). The volume loaded for each sample was normalized with respect to total protein content of the original cell lysate as determined via Bradford assay, in order to control for any variations in cell growth. PolyP was visualized via negative 4′,6-diamidino-2-phenylindole (DAPI) staining (53).

### Biofilm assay.

P. aeruginosa biofilms were quantified as described previously (54). Briefly, M63 medium supplemented with 1 mM MgSO_4_ and 0.4% arginine as the sole carbon source was colony inoculated with PA14. Biofilms were allowed to form in 96-well tissue culture plates (U-bottom; Sarstedt) at 37°C for 16 h. Wells were washed twice with sterile water. Biofilm rings were stained with 0.1% crystal violet, washed twice with water, and then solubilized in 30% acetic acid to record absorbance at 570 nm.

### Swimming motility.

Swimming motility plates consisted of 0.3% (wt/vol) Bacto agar (Becton Dickinson) and 2.5% (wt/vol) LB (Miller; Bioshop). Where indicated, 100 μM compound or an equivalent volume of DMSO was added to the agar prior to pouring. Plates were stab inoculated with a pipette tip dipped in overnight LB culture. Plates were incubated in sealed Tupperware at 30°C for 18 h, and swimming diameter was imaged and measured electronically.

### Pyoverdine and pyocyanin quantification.

Fifty-milliliter P. aeruginosa cultures in 2.5% (wt/vol) LB (Miller; Bioshop) were grown to stationary phase in 125-ml Erlenmeyer flasks with 180-rpm shaking at 37°C for 18 h. Cultures were centrifuged at 18,000 rpm for 30 min to clarify the supernatant, which was then filter sterilized. Pyoverdine was quantified spectrophotometrically at 403 nm as described previously (55). Pyocyanin was extracted via chloroform and treated with HCl to yield a red product that was quantified at 520 nm (55). To account for any colorimetric interference by inhibitors, cell-free blank readings were subtracted from each sample.

### C. elegans infection model.

Wild-type N2 C. elegans and the *glp-4* conditionally sterile mutant strain were obtained from the Caenorhabditis Genetics Center. Liquid killing assays were conducted using *glp-4* mutant worms as described previously (35), with the exception of survival being scored manually under a Zeiss light microscope instead of via Sytox Orange staining. Nematodes were scored as dead when they no longer moved when tapped. Solid killing was conducted using *glp-4* as described previously (34), with the indicated concentration of inhibitor impregnated into the SK agar. Bacterial CFU accumulation in WT N2 worms was quantified as described previously (56). Briefly, age-synchronized embryos were spotted onto bacterium-seeded nematode growth medium (NGM) agar plates impregnated with inhibitor or DMSO. Embryos were incubated at 25°C for 3 days, after which point adult worms were harvested, washed, lysed, and enumerated for CFU.

Assessment of antibiotic synergy in the C. elegans model was adapted from previously described protocols (25). Briefly, *glp-4* C. elegans worms were incubated at 25°C with P. aeruginosa and the indicated amount of gallein or DMSO in LK medium as described above. After 24 h, antibiotics were added. After another 24 h, C. elegans survival was scored as described above.

### HEK293T cytotoxicity assay.

HEK293 cells were grown in Dulbecco’s modified Eagle’s medium supplemented with 10% fetal bovine serum at 5% CO_2_ and 37°C. Cells were grown until a confluent monolayer was formed in 12-well culture plates, at which point inhibitors or DMSO was added. After 48-h incubation, cells were detached with trypsin and percent viability was scored via a hemocytometer and trypan blue staining.
