# Supplementary material for: A Dual-Specificity Inhibitor Targets Polyphosphate Kinase 1 and 2 Enzymes To Attenuate Virulence of Pseudomonas aeruginosa
Source: mBio. 2021 Jun 15;12(3):e00592-21. doi: 10.1128/mBio.00592-21 (PMC8262977; doi:10.1128/mBio.00592-21)
Supplement: TEXT S1 [file mbio.00592-21-s0001.docx]

**A dual-specificity inhibitor targets polyphosphate kinase 1 and 2 enzymes**

**to attenuate virulence of *Pseudomonas aeruginosa***

Nolan Neville,^1^ Nathan Roberge,^1^ Xiang Ji,^2,3^

Preyesh Stephen,^1^ Jiasheng Louis Lu,^2,4^ and Zongchao Jia ^1,*^

^1^ Department of Biomedical and Molecular Sciences, Queen’s University, Kingston, Ontario, Canada

^2^ Risen (Shanghai) Pharma Tech Co., Ltd., Shanghai, China

^3^ Department of Biochemistry, School of Life Sciences, Fudan University, Shanghai, China

^4^ Guangdong Key Laboratory of Nanomedicine, Chinese Academy of Sciences, Shenzhen, China

^*^ Correspondence should be addressed to Z. J. jia@queensu.ca

**SUPPLEMENTAL NOTES AND DISCUSSION**

**Assay selection for PPK polyP synthesis activity**

Several common methods exist for quantifying polyP produced by PPKs, including labelling of polyP via radioactive ATP (1), fluorescent detection via the dye 4',6-diamidino-2-phenylindole (DAPI) (2), or detection via metachromic shift of the dye toluidine blue (3). While radioactive detection is highly sensitive, it is laborious and requires stringent radiation safety precautions. DAPI-based detection of polyP also offers high sensitivity, but the fluorescence signal of this assay is susceptible to fluorescence quenching by other compounds when screening for PPK inhibitors (2). Unfortunately, ellagic acid and several of its derivatives including gallein were strong fluorescence quenchers of DAPI fluorescence in the presence of polyP (data not shown). We therefore selected the toluidine blue assay for this study. Toluidine blue undergoes a metachromic shift from blue to pink colour upon binding polyP, which can be detected via quantification of the A_630 nm_/A_520 nm_ ratio. This method yielded an acceptable linear range of 0-20 µM polyP (in terms of P_i_ monomers) (**Fig. S1A**), allowing for calculation of polyP production based on the standard curve. Any colorimetric interference by the compounds was corrected by subtracting a blank (all ingredients except enzyme) for every sample.

**PPK1 assay conditions**

Since PPK1 activity is strongly inhibited by ADP (K_i_ = 0.09 mM for *E. coli* PPK1) (1), it is an established practice to include an ATP regeneration system consisting of creatine kinase and creatine phosphate in the reaction mix to improve signal quality (4, 1, 2). We included this regeneration system for all IC_50_ and kinetic assays presented, unless otherwise stated. As this introduces another enzyme (creatine kinase), which itself could be inhibited by small molecules, we also assayed PPK1 activity in the absence of creatine kinase/creatine phosphate. Low-micromolar gallein treatment reduced both PPK1 activity rate and equilibrium polyP levels (5) in the absence of ATP regeneration (**Fig. S1C, D**), indicating that gallein activity was due to inhibition of PPK1 and not interreference with creatine kinase.

**SUPPLEMENTAL METHODS**

**Chemical synthesis**

Synthesis of **RT1** (3-hydroxy-4,7,8-trimethoxy-6*H*-benzo[*c*]chromen-6-one)

The titled compound was synthesized according to the procedure of 3-hydroxy-8-methoxy-6*H*-benzo[*c*]chromen-6-one using 2-methoxybenzene-1,3-diol as start material. ^1^H NMR (CDCl_3_, 500 MHz) δ ppm 4.01 (s, 3H), 4.05 (s, 3H), 4.17 (s, 3H), 6.00 (s, 1H), 6.96 (d, *J*=8.8 Hz, 1H), 7.44 (d, *J*=8.9 Hz, 1H), 7.63 (d, *J*=8.8 Hz, 1H), 7.78 (d, *J*=8.8 Hz, 1H). ^13^C NMR (CDCl_3_, 125 MHz) δ ppm 56.56, 61.50, 61.86, 111.51, 111.78, 114.26, 117.22, 117.43, 119.86, 129.54, 133.91, 143.91, 149.59, 151.67, 152.79, 156.91. m/z (ESI^-^) 300.8.

Synthesis of **RT2** (1,2,3,7,8-pentahydroxychromeno[5,4,3-*cde*]chromene-5,10-dione) (6)

A suspension of gallic acid (10 g, 5.9 mmol) in concentrated.H_2_SO_4_ (80 mL) and H_2_O (33 mL) was cooled to -50 ^o^C, then charged with K_2_S_2_O_8_ (20 g, 7.4 mmol). The mixture was stirred at 4 ^o^C for 16 h. The precipitate was filtered and recrystallized from pyridine to afford 1,2,3,7,8-pentahydroxychromeno[5,4,3-*cde*]chromene-5,10-dione (2.0 g, 10.4 %). ^1^H NMR (DMSO-*d_6_,* 500 MHz) δ ppm 7.49 (s, 1H). ^13^C NMR (DMSO-*d_6_*, 125 MHz) δ ppm 93.98, 108.71, 109.51, 110.75, 113.09, 130.92, 135.19, 136.29, 140.02, 142.26, 146.90, 148.68, 159.70, 162.91. m/z (ESI^-^): 316.8.

Synthesis of **RT3/ellagic acid** (2,3,7,8-tetrahydroxychromeno[5,4,3-*cde*]chromene-5,10-dione)

A solution of 3,4,5-trimethoxybenzoic acid (1 g, 4.7 mmol), [Rh(NBD)Cl]_2_ (13.8 mg, 0.03 mmol, 0.6 mol%), and MnO_2_ (1.23 g, 14.1 mmol, 3 equiv) in distilled water (5 mL) was stirred in a sealed tube at 150 ^o^C for 72 h under an atmosphere of air. The reaction mixture was cooled to room temperature and acidified to pH<3 with 2 M HCl, and concentrated. The residue was purified by column chromatography (MeOH/DCM=1/20~1/5) to afford 4,4',5,5',6,6'-hexamethoxy-[1,1'-biphenyl]-2,2'-dicarboxylic acid (702 mg, 35.3%). BBr_3_ (2 mL, 21.5 mmol) was added dropwise to the solution of 4,4',5,5',6,6'-hexamethoxy-[1,1'-biphenyl]-2,2'-dicarboxylic acid (108 mg, 0.25 mmol) in DCM (25 mL) at 0 ^o^C under nitrogen atmosphere. The mixture was allowed to slowly warm to room temperature, after which it was stirred for an additional 40 h at room temperature. Then the mixture was slowly poured into ice, filtered and washed with cold methanol. The solid was dried in vacuum to afford 2,3,7,8-tetrahydroxychromeno[5,4,3-*cde*]chromene-5,10-dione (75 mg, 99.3%). ^1^H NMR (DMSO-*d_6_*, 500 Hz), δ ppm 7.46 (s, 2H), 10.55 (s, 2H), 10.80 (s, 2H). ^13^C NMR (DMSO-*d_6_*, 125 Hz), δ ppm 107.71, 110.22, 112.34, 136. 53, 139.47, 148.11, 159.20. m/z (ESI^-^) 301.1.

Synthesis of **RT4/gallein** (7)

ZnO (81.4 mg, 1.0 mmol) was added to a mixture phthalic anhydride (1.5 g, 10 mmol) and pyrogallol (2.5 g, 20 mmol), and heated to 160 ^o^C for 2 h, then cooled to room temperature. The mixture was dissolved in 1 N NaOH (10 mL) and filtered. The filtrate was acidified to pH=7, and the precipitation was filtered. The solid was recrystallized with MeOH and dried to afford gallein (2.2 g, 61.1%). ^1^H NMR (DMSO-*d_6_*, 500 MHz), δ ppm 6.01 (d, *J*=7.5 Hz, 2H), 6.58 (d, *J*=7.5 Hz, 2H), 7.29 (dd, *J_1_*=1.5Hz, *J_2_*=7.5 Hz, 1H), 7.71-7.79 (m, 2H), 7.98 (dd, *J_1_*=1.5Hz, *J_2_*=7.5 Hz, 1H), 9.31 (bs, 4H). ^13^C-NMR (DMSO-*d_6_*, 125 MHz), δ ppm 83.93, 110.43, 112.17, 116.76, 124.01, 124.41, 126.09, 129.89, 132.78, 135.23, 140.10, 146.38, 152.07, 168.61. m/z (ESI^+^) 363.0.

**Molecular docking.** Docking was conducted in Molecular Operating Environment (MOE) (Chemical Computing Group, Montreal, Canada). Protein homology models were energy minimized, protonated, then checked for structural issues using QuickPrep. Gallein was then docked to the protein using the rigid receptor refinement setting to select the top-scoring pose.

**SUPPLEMENTAL REFERENCES**

1. Ahn K, Kornberg A. 1990. Polyphosphate kinase from Escherichia coli. Purification and demonstration of a phosphoenzyme intermediate. J Biol Chem 265:11734–11739.

2. Dahl J-U, Gray MJ, Bazopoulou D, Beaufay F, Lempart J, Koenigsknecht MJ, Wang Y, Baker JR, Hasler WL, Young VB, Sun D, Jakob U. 2017. The anti-inflammatory drug mesalamine targets bacterial polyphosphate accumulation. Nat Microbiol 2:16267.

3. Candon HL, Allan BJ, Fraley CD, Gaynor EC. 2007. Polyphosphate Kinase 1 Is a Pathogenesis Determinant in Campylobacter jejuni. J Bacteriol 189:8099–8108.

4. Bravo-Toncio C, Álvarez JA, Campos F, Ortíz-Severín J, Varas M, Cabrera R, Lagos CF, Chávez FP. 2016. Dictyostelium discoideum as a surrogate host-microbe model for antivirulence screening in Pseudomonas aeruginosa PAO1. Int J Antimicrob Agents 47:403–409.

5. Rudat AK, Pokhrel A, Green TJ, Gray MJ. 2018. Mutations in Escherichia coli Polyphosphate Kinase That Lead to Dramatically Increased In Vivo Polyphosphate Levels. J Bacteriol 200:e00697-17.

6. Cozza G, Gianoncelli A, Bonvini P, Zorzi E, Pasquale R, Rosolen A, Pinna LA, Meggio F, Zagotto G, Moro S. 2011. Urolithin as a converging scaffold linking ellagic acid and coumarin analogues: design of potent protein kinase CK2 inhibitors. ChemMedChem 6:2273–2286.

7. Jadhav NH, Shinde DR, Sakate SS, Rasal NK, Pawar RA. 2019. Ti(IV) doping: An effective strategy to boost Lewis acidic performance of ZnO catalyst in fluorescein dye synthesis. Catal Commun 120:17–22.
