# Supplementary figures and images for: A Dual-Specificity Inhibitor Targets Polyphosphate Kinase 1 and 2 Enzymes To Attenuate Virulence of Pseudomonas aeruginosa
Source: mBio. 2021 Jun 15;12(3):e00592-21. doi: 10.1128/mBio.00592-21 (PMC8262977; doi:10.1128/mBio.00592-21)

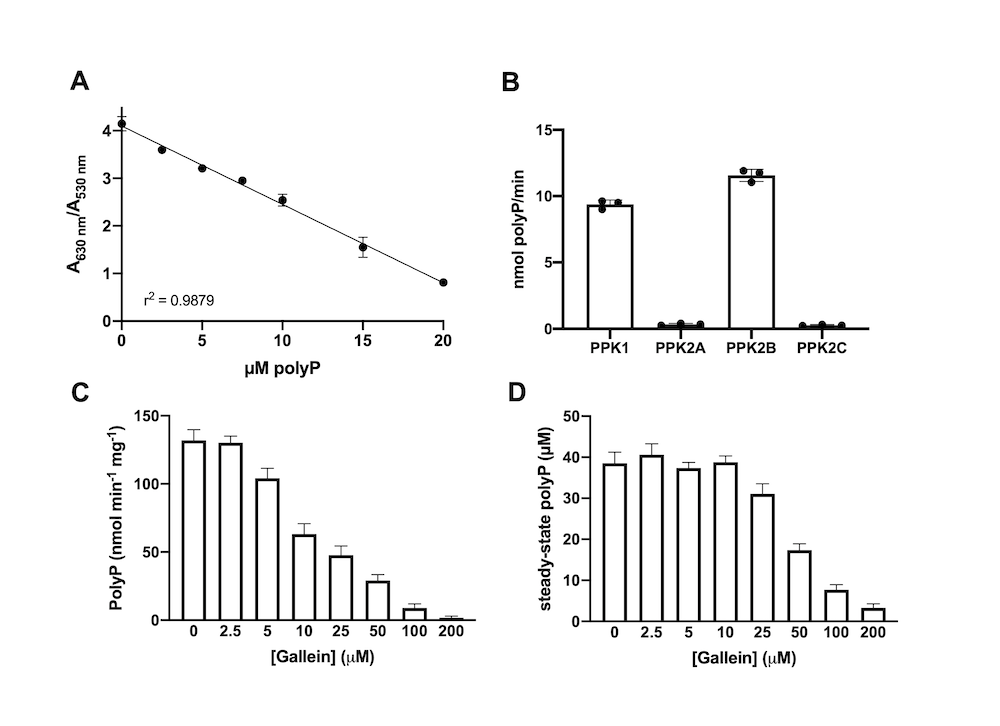

Supplement: FIG S1 [file mbio.00592-21-sf001.tif]

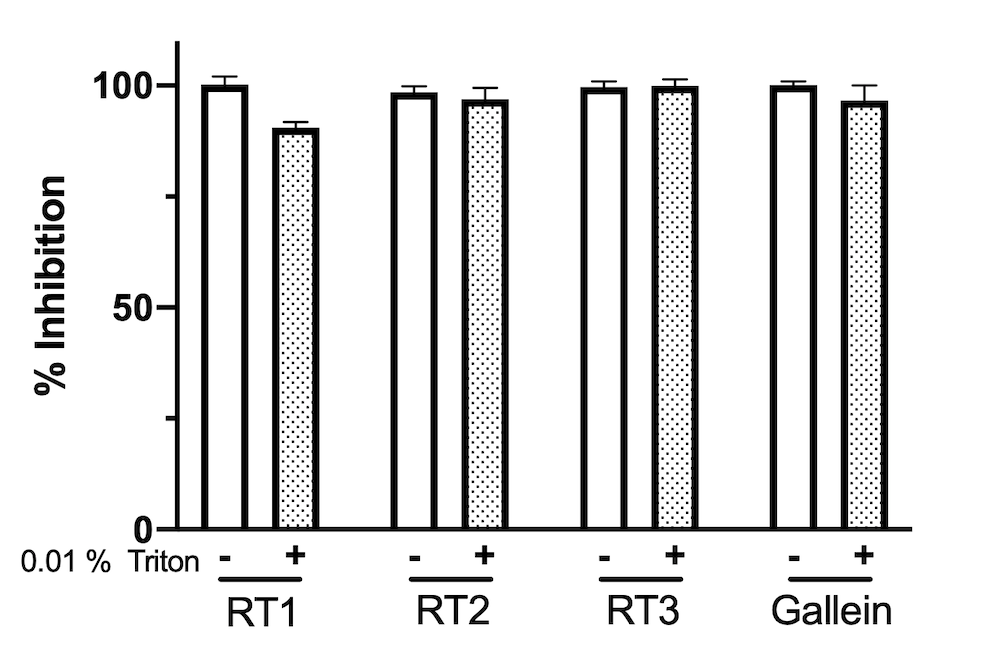

Supplement: FIG S2 [file mbio.00592-21-sf002.tif]

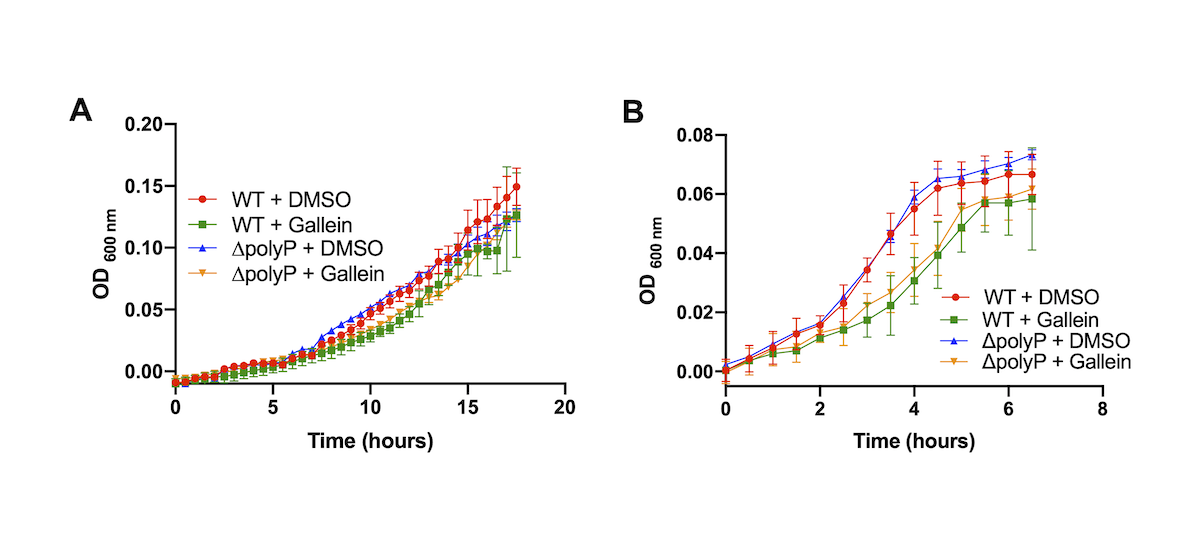

Supplement: FIG S4 [file mbio.00592-21-sf004.tif]
