# Supplementary material for: A Dual-Specificity Inhibitor Targets Polyphosphate Kinase 1 and 2 Enzymes To Attenuate Virulence of Pseudomonas aeruginosa
Source: mBio. 2021 Jun 15;12(3):e00592-21. doi: 10.1128/mBio.00592-21 (PMC8262977; doi:10.1128/mBio.00592-21)
Supplement: TABLE S1 [file mbio.00592-21-st001.docx]

**Table S1: Strains, plasmids, and oligos used in this study**

| **Strains** | | | | | | |
| --- | --- | --- | --- | --- | --- | --- |
| **Strain name** | | | **Genotype** | | **Source** | |
| ***E. coli* strains** | | | | | | |
| BL21 (DE3) | | | fhuA2 [lon] ompT gal (λ DE3) [dcm] ∆hsdS λ DE3 = λ sBamHIo ∆EcoRI-B int::(lacI::PlacUV5::T7 gene1) i21 ∆nin5 | | New England Biolabs | |
| OP50 | | |  | | Available from CGC, University of Minnesota | |
| ***P. aeruginosa* strains** | | | | | | |
| WT | | | *P. aeruginosa* UCBPP-PA14 | | (1) | |
| *∆ppk1* | | | *P. aeruginosa* UCBPP-PA14 *∆ppk1*; deletion of PA14_69230 | |  |  |
| *∆ppk2A ∆ppk2B ∆ppk2C* | | | *P. aeruginosa* UCBPP-PA14 *∆ppk2A ∆ppk2B ∆ppk2C*; deletion of PA14_01730, PA14_33240, and PA14_19410 | |  |  |
| *∆polyP* | | | *P. aeruginosa* UCBPP-PA14 *∆ppk1* *∆ppk2A ∆ppk2B ∆ppk2C*; deletion of PA14_69230, PA14_01730, PA14_33240, and PA14_19410 | |  |  |
| ***C. elegans* strains** | | | | | | |
| N2 | | | N2 wildtype | | Available from CGC, University of Minnesota | |
| *glp-4* | | | *glp-4 (bn2)* | |  |  |
| **Plasmids** | | | | | | |
| **Plasmid name** | **Purpose** | | | **Antibiotic** | | **Source** |
| HT29 | Expression vector based on pET16 with N-His6-MBP-TEV-MCS | | | Amp^R^ | | This study |
| PPK1 pET-TEV | *ppk1* expression | | | Kan^R^ | | (2) |
| NN50 | *ppk2A* expression; HT29 derivative | | | Amp^R^ | | This study |
| NN23 | *ppk2B* expression; HT29 derivative | | | Amp^R^ | | This study |
| NN56 | *ppk2C* expression; HT29 derivative | | | Amp^R^ | | This study |
| **Oligos** | | | | | | |
| **Oligos name** | | **Sequence** | | | | |
| PA_PPK2A_A8_Fwd | | atatGGATCCCCCCACGGCAGCAGCGAGGAC | | | | |
| PA_PPK2A_P289_Rev | | atatCTCGAGTCACGGCGCGGCGGCCGC | | | | |
| PPK2B_MBP_Fwd | | atatGGATCCATGGACTCCTATGGCGATACC | | | | |
| PPK2B_MBP_Rev | | atatCTCGAGTCAATAGACTTCCGGCACG | | | | |
| PA_PPK2C_Fwd | | atatCATATGATGTTCGAATCCGCGGAAG | | | | |
| PA_PPK2C_Rev | | atatGAATTCTCACTTGTCCTTCTTGTACGCC | | | | |

**REFERENCES**

1. Racki LR, Tocheva EI, Dieterle MG, Sullivan MC, Jensen GJ, Newman DK. 2017. Polyphosphate granule biogenesis is temporally and functionally tied to cell cycle exit during starvation in Pseudomonas aeruginosa. Proc Natl Acad Sci USA 114:E2440–E2449.

2. Bravo-Toncio C, Álvarez JA, Campos F, Ortíz-Severín J, Varas M, Cabrera R, Lagos CF, Chávez FP. 2016. Dictyostelium discoideum as a surrogate host-microbe model for antivirulence screening in Pseudomonas aeruginosa PAO1. Int J Antimicrob Agents 47:403–409.
